# The Image Quality and Diagnostic Performance of CT with Low-Concentration Iodine Contrast (240 mg Iodine/mL) for the Abdominal Organs

**DOI:** 10.3390/diagnostics12030752

**Published:** 2022-03-19

**Authors:** Moon-Hyung Choi, Young-Joon Lee, Seung-Eun Jung

**Affiliations:** Department of Radiology, Eunpyeong St. Mary’s Hospital, College of Medicine, The Catholic University of Korea, Seoul 03312, Korea; cmh@catholic.ac.kr (M.-H.C.); sejung@catholic.ac.kr (S.-E.J.)

**Keywords:** contrast media, computed tomography, iodine concentration: image quality, enhancement

## Abstract

Purpose: To evaluate the difference between CT examinations using 240 mgI/mL contrast material (CM) and 320 mgI/mL CM in the contrast enhancement of the abdominal organs and the diagnostic performance for focal hepatic lesions. Materials and methods: This retrospective study included 422 CT examinations, using 240 mgI/mL iohexol (Group A, 206 examinations) and 320 mgI/mL ioversol (Group B, 216 examinations), performed between April 2019 and May 2020. Two CT scanners (single-source CT (machine A) and dual-source CT (machine B)) were used to obtain CT images. Two radiologists independently drew regions of interest (ROIs) in the liver, pancreas, spleen, kidney, aorta, portal vein, and paraspinal muscle. The signal-to-noise ratio (SNR) and contrast-to-noise ratio (CNR) were calculated for each organ. They evaluated the degree of subjective enhancement of the organs and detected/differentiated focal hepatic lesions. Results: The SNR, CNR, and subjective enhancement of most organs were significantly higher in Group B than in Group A (*p* < 0.05). The sensitivity and specificity for cysts and malignancy were higher than 85.0% in both groups. The sensitivity for hemangioma was lower in Group B (<75%) than in Group A. In Group A, the SNR and CNR were significantly higher in most organs with machine B than with machine A. Conclusion: Although the SNR and CNR of the abdominal organs were lower with 240 mgI/mL CM than with 320 mgI/mL CM, 240 mgI/mL CM was feasible for evaluating the liver. A CT scanner with more advanced specifications may be beneficial for examinations with 240 mgI/mL CM by using lower tube voltage.

## 1. Introduction

CT examinations are widely used for diagnosis or patient follow-up. As the diagnostic performance of contrast-enhanced CT is superior to that of unenhanced CT, intravenous iodinated contrast material (CM) is frequently used. A disadvantage of using iodine CM is the risk of contrast-induced nephropathy (CIN) [1,2,3]. Even though the incidence of CIN was low in patients with normal renal function or mild renal insufficiency [4,5,6], elevated serum creatinine levels before CT examination and known renal disease are risk factors for CIN due to intravenous iodine CM [5,7,8]. CIN is associated with increased mortality, and a high cumulative volume of iodine CM for patients with acute kidney injury was associated with poor renal outcome [9,10]. Moreover, the iodine load of patients is associated with the risk of CIN [11,12]. Therefore, the smallest possible dose of CM is recommended to minimize the risk of CIN [1,3,13].

We can reduce the total amount of CM or the concentration of CM to decrease the total amount of iodine. Several studies have shown the difference in hepatic enhancement between two concentrations of CM. Comparable enhancement was observed in the liver with two different concentrations (300 mgI/mL vs. 370 mgI/mL) of the same CM when the total iodine load was fixed [14,15,16]. However, other studies showed that mean hepatic enhancement was significantly better with 370 mgI/mL CM than with 300 mgI/mL CM when the total volume of CM was fixed [17,18]. In the pancreas, a higher dose of CM increased the maximum enhancement [19]. It seems that enhancement is affected by the total amount of iodine rather than the concentration of CM. However, radiologists may hesitate to use CM with a lower iodine concentration because the reduction in iodine load may affect diagnostic performance. Some studies showed that 240 mgI/mL CM was feasible for CT urography [20,21]. We questioned to what extent the use of 240 mgI/mL CM affected the contrast enhancement of the abdominal organs in routine abdominal CT. The purpose of this study was to evaluate the difference between CT examinations using 240 mgI/mL CM and 320 mgI/mL CM in the enhancement of the abdominal organs and the diagnostic performance for focal hepatic lesions.

## 2. Materials and Methods

### 2.1. Subjects

As the feasibility of 240 mgI/mL CM had not yet been proven for abdominal CT many organs with similar density, we could not use 240 mgI/mL CM for abdominal CT. However, 240 mgI/mL CM had been used for chest CT in our institution because the lung and mediastinal structures have very obvious contrast. Therefore, we evaluated the abdominal organs included in chest CT. Chest CT examinations performed between April 2019 and May 2020 were eligible for inclusion in this study. To cross-compare the image quality according to the CM and CT equipment, we searched patients with CT scans using different CMs (240 mgI/mL iohexol (Iobrix 240, Taejoon pharm., Seoul, Korea) and 320 mgI/mL ioversol (Optiray 320, Guerbet, Villepinte, France)) and the same machine or CT scans performed on different machines with the same CM. Both iohexol and ioversol are low osmolar CM. CT examinations were divided into two groups according to the concentration of the CMs: Group A with 240 mgI/mL and Group B with 320 mgI/mL. A total of 422 CT examinations (Group A: 206 examinations, Group B: 216 examinations) were included in this study. Clinical information such as age, sex, body weight, and height were collected from electronic medical records. Radiation dose-related information, including tube voltage, tube current, volume computed tomography dose index (CTDIvol) and dose length product (DLP), was collected from CT dose reports. The volume of CM that was used in each examination was recorded in the examination. We collected the information to calculate the amount of iodine.

### 2.2. CT Protocol

CT examinations were performed with two different CT machines: “machine A” was a 128-slice single-source CT scanner (Somatom Edge, Siemens Healthineers, Erlangen, Germany) with available tube potential 70–140 kVp and 20–800 mA, and “machine B” was a dual-source 384-slice (2 × 192) CT (Somatom Force, Siemens Healthineers, Erlangen, Germany) with available tube potential 70–150 kVp and 20–1300 mA. CT images were obtained 55 s after CM injection (1.4 mL/kg, 2 mL/sec) and 20 mL saline flush with a power injector (Medrad injector, Medrad, Warrendale, PA, USA) via the antecubital vein. As the same amount of CM per kg was used for both CMs, the iodine amount per kg in groups A and B was 336 mg/kg and 448 mg/kg. The acquisition parameters were similar for both machines: slice thickness 3 mm with a 3-mm interval; rotation time, 0.5 s; pitch, 1; automated tube voltage modulation (CARE kV, Siemens Healthineers) with reference kV 120; automatic tube current selection (CAREDose 4D, Siemens Healthineers) with reference mAs 130; collimation 128 × 0.6 for machine A and 192 × 0.6 for machine B.

### 2.3. Image Analysis

**Figure 1:**
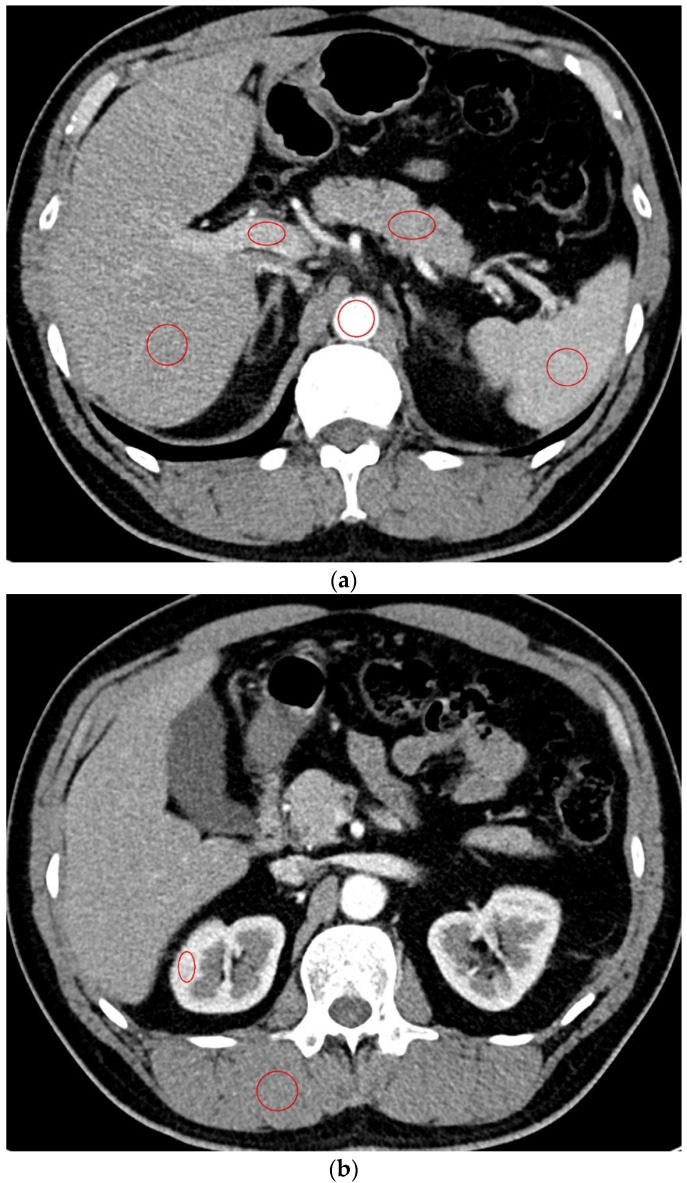
Quantitative analysis of the CT images A radiologist draws multiple regions of interest in the liver, pancreas, spleen, portal vein, and aorta (**a**) and in the kidney and paraspinal muscle (**b**).

Two radiologists with more than 10 years of experience with abdominal radiology independently evaluated the chest CT images that were reconstructed with soft kernel (Br40). For quantitative analysis, they identified seven regions of interest (ROIs) in the liver, pancreas, spleen, kidney, aorta, portal vein, and paraspinal muscle. It was recommended that the size of the ROI be 2 cm^2^ or larger in the liver, spleen, and paraspinal muscle and as large as possible in the other organs on the single axial image that contained the largest area of each organ (Figure 1). They drew ROIs in the renal cortex avoiding the medulla to evaluate the kidney and in the paraspinal muscle area that showed the most homogeneous density. From the ROI, the mean density and standard deviation (aka noise) were extracted. The signal-to-noise ratio (SNR) was calculated as the mean density/standard deviation in each organ. The contrast-to-noise ratio (CNR) relative to muscle was calculated as (mean density of the organ–mean density of the paraspinal muscle)/standard deviation of the paraspinal muscle.

The same radiologists also performed a qualitative image analysis without any information about the CT parameters or clinical data. They subjectively evaluated the degree of contrast enhancement in the liver, pancreas, spleen, portal vein, aorta, and kidney and the overall noise of the images using a 5-point scale (1, very poor; 2, poor; 3, moderate; 4, good; 5, excellent). The two radiologists independently determined the presence of focal hepatic lesions and differentiated the lesions among cysts, hemangiomas, and malignancies. The final diagnosis was determined after discrepancies were resolved by consensus reading. They also predicted which CM would have been used for each examination.

### 2.4. Statistical Analysis

The interobserver agreement between the two radiologists who assessed the quantitative and qualitative analysis results was evaluated with intraclass correlation coefficient (ICC).

The patient’s characteristics and CT-related factors, such as radiation exposure and the amount of iodine, were compared between Groups A and B using the Fisher’s exact tests for categorical variables and the Student’s t-test for continuous variables. The mean values of the two readers were used to assess the quantitative and qualitative analysis results. The differences in the quantitative and qualitative parameters between the two groups were analyzed using the Student’s t-test. Additionally, the differences in the image quality between the CT machines and tube voltage (≤90 kVp vs. ≥100 kVp) in each group were evaluated using the Student’s t-test.

The interobserver agreement between the two radiologists, regarding the diagnosis, was analyzed for the entire examination and the two groups using the Kappa values. The sensitivity and specificity were calculated to detect the lesions. The agreement between the CM that was estimated by the radiologist and the CM that was used was also analyzed by the Kappa values. The Kappa value and ICC were interpreted as follows: <0.40, poor; 0.40≤ and <0.60, fair; 0.60≤ and <0.80, good; and 0.81–1.00, excellent agreement.

The institutional review board of our hospital approved this study and waived the requirement for informed consent due to the retrospective study design.

## 3. Results

There were no significant differences in the patients’ characteristics and the CT examinations between the two groups (Table 1). Tube voltage and CTDIvol were significantly higher in Group A than Group B.

The interobserver agreement for the mean density, which was a factor of quantitative analysis, and subjective degree of enhancement in the abdominal organs is summarized in Table 2. Quantitative analysis showed good agreement in all the organs except the portal vein, which showed poor agreement. The interobserver agreement, for the subjective degree of enhancement in the liver, portal vein, aorta, and kidney, was good.

In the quantitative analysis, the SNR was significantly higher in the spleen, portal vein, aorta, and the kidney in Group B than in Group A (Table 3). The CNR of all the organs except the liver was higher in Group B than in Group A. Subjective enhancement in all the organs was higher in Group B than in Group A (Table 4). The subjective noise level was not different between the two groups.

**Figure 2:**
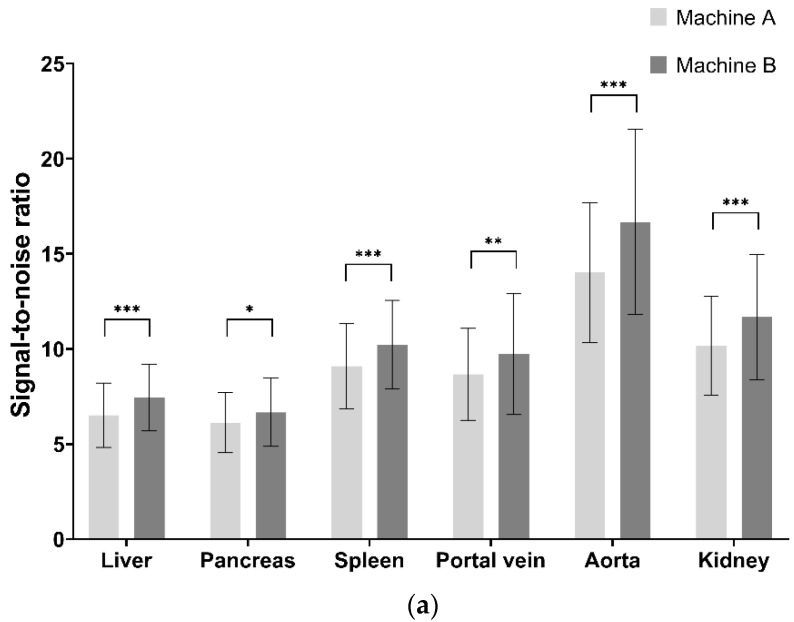

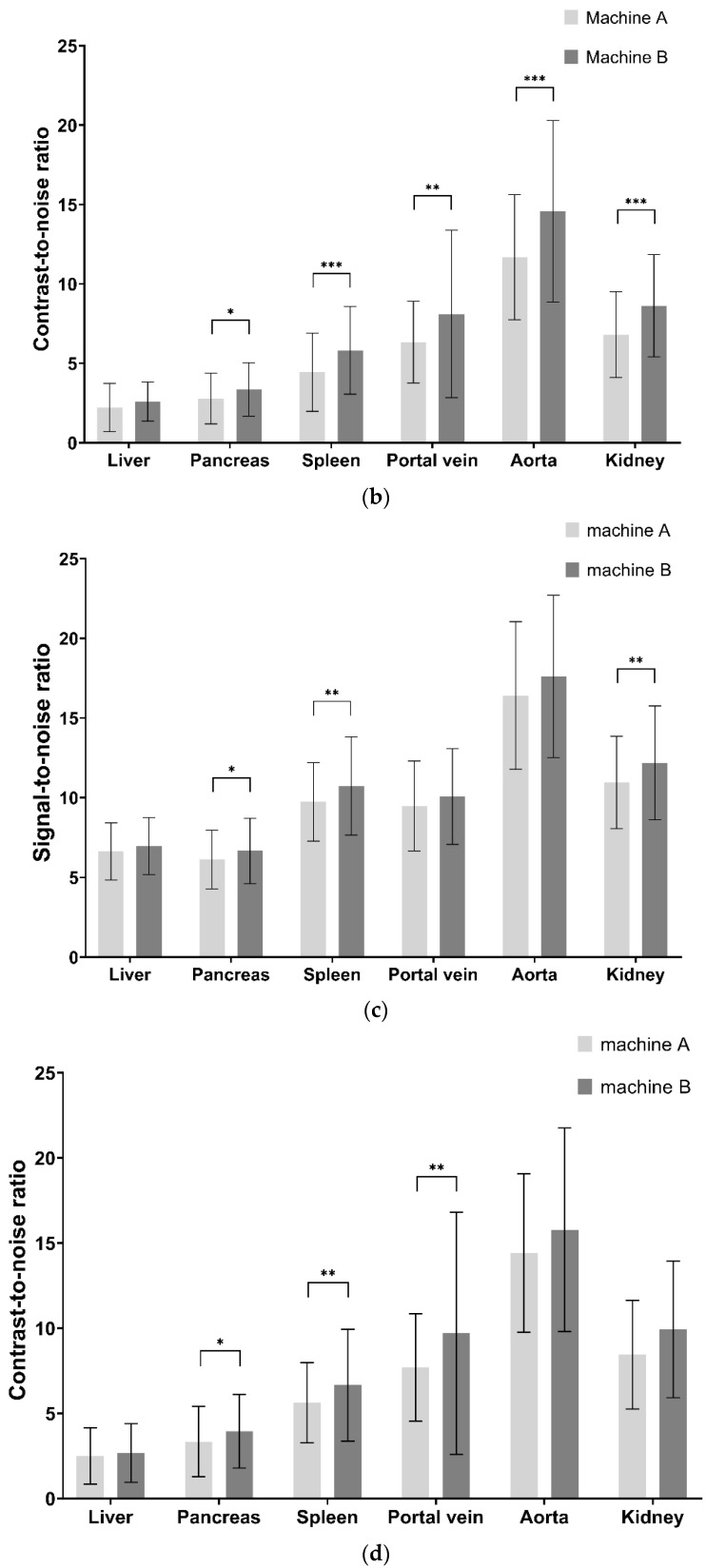
Differences in the signal-to-noise ratio (SNR) and contrast-to-noise ratio (CNR) between the two machines in each group SNR and CNR of the organs in Group A (**a**,**b**) and Group B (**c**,**d**), respectively. * *p* < 0.05; ** *p* < 0.01; *** *p* < 0.001.

In each group, some quantitative parameters were significantly different between the machines that performed CT examinations (Figure 2). In Group A, the SNR of all the organs and the CNR of all the organs except the liver were significantly higher with machine B than machine A. In Group B, the SNR of the pancreas, spleen, and kidney and the CNR of the pancreas, spleen, and portal vein were significantly higher with machine B than machine A. The machine A frequently selected tube voltage 100 kVp or higher than machine B with statistical significance (Group A: 93.6% vs. 47.4%, *p* < 0.001; Group B: 83.3% vs. 40.2%, *p* < 0.001). All quantitative analysis parameters were significantly higher in CT scans with lower tube voltage than those with higher tube voltage in both groups (Table 5).

**Figure 3:**
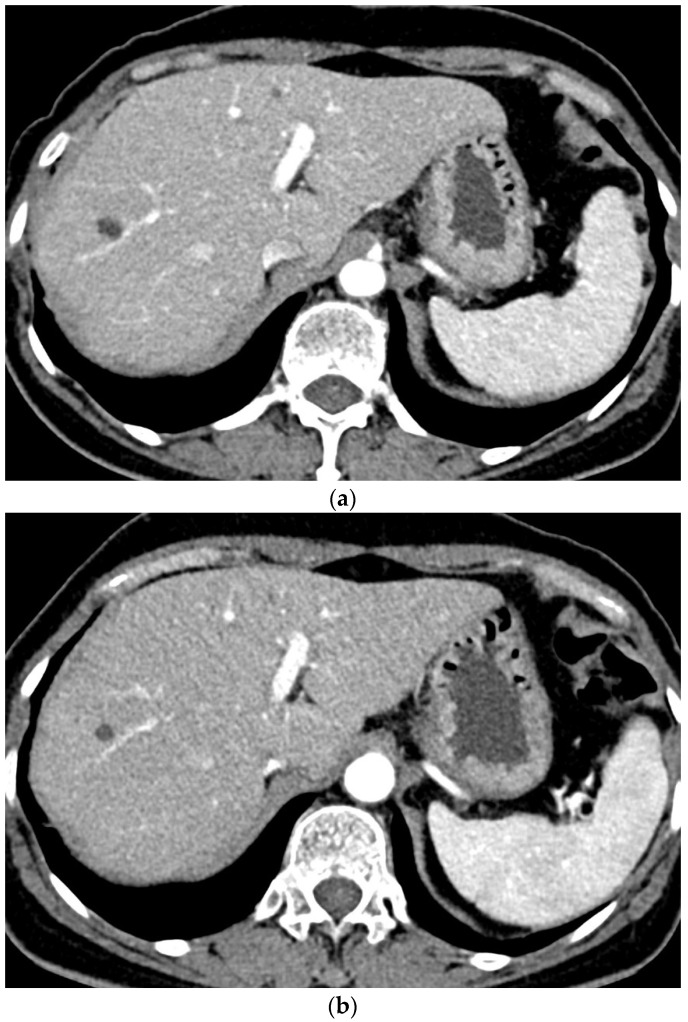
A 64-year-old female patient who underwent CT examinations using the same machine (machine B). An approximately 0.7-cm cyst is noted in liver segment 8 on both CT examinations with 240 mgI/mL contrast material (**a**) and 320 mgI/mL contrast material (**b**).

A total of 212 focal hepatic lesions, including 134 cysts, 25 hemangiomas, and 53 malignant lesions, were detected in all the examinations. The interreader agreement was fair to excellent for three diagnoses. The Kappa values between the two radiologists to detect cyst, hemangioma, and malignancy were 0.802, 0.712, and 0.935 in all the examinations, 0.796, 0.808, and 0.905 in Group A and 0.808, 0.610, and 0.923 in Group B. Compared to the gold standard, sensitivity was lowest for the hemangiomas by both readers in Group B (Table 6). Sensitivity to detect cysts was lower by reader 1 and higher by reader 2 in Group A than in Group B. Specificity was higher than 98% for three diagnoses by both readers. CT images of the representative patient who underwent CT scans using two different CMs are shown in Figure 3.

Reader 1 and reader 2 correctly guessed the CM in 42.2% (87/216) and 39.4% (85/216) of examinations in Group A and 80.6% (174/216) and 80.1% (173/216) in Group B.

## 4. Discussion

The feasibility of the low-iodine-concentration (240 mgI/mL) CM for the abdominal organs was evaluated in this study. The SNR and CNR of many abdominal organs were significantly higher in the CT examinations with 320 mgI/mL CM (Group B) than in those with 240 mgI/mL CM (Group A). Qualitative analysis also showed similar results that subject enhancement of the organs was significantly higher in Group B than Group A. As we used the same volume of CM per kg with different concentrations, which means the different iodine amounts per kg, in the two groups, the differences in the enhancement of the abdominal organs were noted as previously reported [17,18]. Low-iodine-concentration CM may have influenced the detection of hepatic focal lesions, but there was no difference in this study. Even though the SNR and CNR of most organs were higher in Group B than in Group A, the liver SNR and CNR were not different between the two groups, which may be the reason for the lack of significant differences in the diagnostic performance for the liver lesion.

Sensitivity and specificity were acceptable for the cyst, hemangioma, and malignancy in both groups. Rather, the sensitivity of hemangioma was lower in Group B than in Group A for both readers. The timing of a CT scan and a multiphase CT is important to accurately detect hemangiomas in the liver. Meanwhile, a decrease in enhancement may affect the detection of focal lesions such as hepatocellular carcinoma, and arterial enhancement is important. However, it may have less effect on the detection of low-density lesions that were mainly analyzed in this study. Therefore, it seems that low-concentration contrast agents can be used in patients who frequently undergo follow-up CT examinations for malignancy except for hepatocellular carcinoma, in which arterial enhancement is important.

As we did not adjust the CT parameters for the low-iodine-concentration CM, more prominent enhancement by using a lower tube voltage could not be achieved in this study. In a study of 270 mgI/mL CM in the liver, using a lower tube voltage (80 kVp) improved hepatic enhancement [22]. Usually, low tube voltage and advanced reconstruction techniques, such as iterative reconstruction, are used to compensate for the weak enhancement of low-concentration CM or to decrease iodine load [23,24]. Although there was no study that evaluated the image quality of the abdominal organs with 240 mgI/mL CM, several studies showed the feasibility of 240 mgI/mL CM in CT angiography and CT urography [20,21,25]. In these studies, better or comparable image quality was observed in the iteratively reconstructed CT examinations with low-tube-voltage and low-iodine-concentration CM than conventional CT examinations. In this study, as we did not change any CT parameters for the examinations in Group A, the SNR or CNR of most organs was different between the two groups. However, the subjective level of noise was not different between the two groups.

We evaluated the accuracy rate between the concentration of CM that radiologists predicted to have used in the CT and the actual concentration of CM used in the CT. Even though quantitative and qualitative analysis showed significant differences in many parameters between the CT images with 240 mgI/mL CM and 320 mgI/mL CM, radiologists could correctly estimate the CM only in approximately 40% in Group A. Both radiologists assumed that 320 mgI/mL would be used in more than half of the CT examinations with 240 mgI/mL. These results showed that 240 mgI/mL can obtain a level of contrast enhancement that can be recognized similarly to that of 320 mgI/mL CM.

In this study, we also analyzed the difference in image quality between the machines. The SNR and CNR were significantly improved in the CT examinations with machine B compared with machine A, especially for CT scans with 240 mgI/mL. The wider range of available tube voltage and tube current of machine B than machine A and differences in the detector may be the reason for the significant differences between the two machines. The differences in the image quality according to the CT machine have been reported previously [26,27,28,29]. Although automatic tube voltage selection and automatic tube current modulation could affect the image quality, both techniques were applied with similar settings in both machines in this study [29,30,31]. However, a more specific analysis of the difference between the two machines revealed that machine B chose lower kVp (≤90 kVp) with a significantly higher frequency than machine A. Additionally, SNR and CNR were significantly higher in CT examinations with lower kVp than with higher kVp, similar to previous studies [23,32,33,34]. We infer from these results that the CT with more advanced specifications, including advanced tubes and detectors, may be beneficial to improve the image quality of the CT with low-iodine-concentration CM by using lower kVp frequently.

There are several limitations in this study. First, we used chest CT in this study. As contrast-enhanced chest CT and abdominal CT are usually obtained with different time delays after CM injection, chest CT images cannot completely reflect abdominal CT. However, as we used the images that were reconstructed using the same soft tissue kernel that was used for the abdominal CT, the texture of the CT images was similar to that of the abdominal CT images. Second, we did not change the CT parameters to improve the enhancement when we used 240 mgI/mL. Many previous studies used low tube voltage to compensate for low-concentration CM [23,32,33,34]. Nevertheless, since various tube voltages were used in CT examinations by the automatic tube voltage selection, we could compare the differences in image quality according to tube voltage A prospective study is helpful to show the change in the quantitative parameters from the combination of low-iodine-concentration CM and low tube voltage in portal phase abdominal CT. Third, we evaluated diagnostic performance only in the liver. We cannot guarantee that detecting focal lesions in other abdominal organs, such as the pancreas and kidneys, is not affected by the concentration of CM. This should be revealed in future studies.

In conclusion, although the SNR and CNR of the abdominal organs were lower with 240 mgI/mL CM than with 320 mgI/mL CM, 240 mgI/mL CM was feasible for evaluating the liver. A CT scanner with more advanced specifications may be beneficial for examinations with 240 mgI/mL CM.

## Figures and Tables

**Table 1:** Baseline characteristics of the patients.

	All Examinations	Group A	Group B	*p* Value
Age (years)	65.9 ± 12.4	60.1 ± 12.0	65.8 ± 12.7	0.821
Height (cm)	160.7 ± 9.1	160.9 ± 9.3	160.4 ±8.9	0.573
Weight (kg)	59.1 ± 12.5	59.2 ± 12.7	59.1 ± 12.3	0.962
CT machine (n)				
Somatom Edge	223	109	114	0.978
Somatom Force	199	90	109	
Tube voltage (kVp)	97.2 ± 8.9	98.3 ± 8.4	96.1 ± 9.2	0.011
Tube voltage ≤ 90 kVp	138	58	80	0.062
Tube current (mAs)	163.6 ± 54.2	163.9 ± 45.6	163.3 ± 61.3	0.907
CTDIvol (mGy)	5.8 ± 2.0	6.0 ± 2.0	5.5 ± 2.1	0.014
DLP (mGy cm)	214.7 ± 84.6	221.2 ± 81.4	208.6 ± 87.4	0.126
Iodine amount (g)	25.6 ± 5.0	23.2 ± 3.9	27.9 ± 4.9	<0.001

CTDIvol, volume CT dose index; DLP, dose length product.

**Table 2:** Interreader agreement (intraclass correlation coefficient with 95% confidence interval) in the quantitative and qualitative image analysis parameters.

	Quantitative Analysis: Mean Density	Qualitative Analysis: Enhancement
Liver	0.763 (0.713–0.805)	0.674 (0.602–0.731)
Pancreas	0.673 (0.604–0.703)	0.485 (0.376–0.574)
Spleen	0.758 (0.706–0.800)	0.490 (0.382–0.579)
Portal vein	0.266 (0.112–0.394)	0.802 (0.761–0.837)
Aorta	0.753 (0.701–0.796)	0.657 (0.585–0.717)
Kidney	0.671 (0.602–0.728)	0.680 (0.613–0.736)

**Table 3:** Quantitative image analysis in both groups according to the concentration of contrast material.

	Group A (240 mgI/mL)	Group B (320 mgI/mL)	*p* Value
SNR			
Liver	7.0 ± 1.8	6.8 ± 1.8	0.324
Pancreas	6.4 ± 1.7	6.4 ± 1.9	0.958
Spleen	9.6 ± 2.4	10.2 ± 2.8	0.022
Portal vein	9.2 ± 2.8	9.8 ± 2.9	0.036
Aorta	15.3 ± 4.5	17.0 ± 4.9	<0.001
Kidney	10.9 ± 3.0	11.5 ± 3.3	0.033
CNR			
Liver	2.4 ± 1.4	2.6 ± 1.7	0.221
Pancreas	3.1 ± 1.7	3.6 ± 2.1	0.002
Spleen	5.1 ± 2.7	6.1 ± 2.9	<0.001
Portal vein	7.2 ± 4.2	8.6 ± 5.5	0.002
Aorta	13.0 ± 5.1	15.1 ± 5.3	<0.001
Kidney	7.7 ± 3.1	9.1 ± 3.7	<0.001

SNR, signal-to-noise ratio; CNR, contrast-to-noise ratio.

**Table 4:** Qualitative analysis in both groups according to the concentration of contrast material.

	Group A (240 mgI/mL)	Group B (320 mgI/mL)	*p* Value
Subjective enhancement			
Liver	4.5 ± 0.6	4.8 ± 0.4	<0.001
Pancreas	4.9 ± 0.3	5.0 ± 0.2	<0.001
Spleen	4.9 ± 0.3	5.0 ± 0.2	<0.001
Portal vein	4.3 ± 0.7	4.6 ± 0.6	<0.001
Aorta	4.9 ± 0.3	4.9 ± 0.2	<0.001
Kidney	4.6 ± 0.5	4.9 ± 0.3	<0.001
Noise	3.6 ± 0.6	3.7 ± 0.5	0.225

**Table 5:** Differences in quantitative image analysis between lower and higher tube voltage.

	Group A (240 mgI/mL)			Group B (320 mgI/mL)		
	≤90 kVp	≥100 kVp	*p* Value	≤90 kVp	≥100 kVp	*p* Value
SNR						
Liver	7.8 ± 2.1	6.6 ± 1.5	<0.001	7.1 ± 2.0	6.6 ± 1.6	0.043
Pancreas	7.0 ± 2.1	6.1 ± 1.5	0.004	6.9 ± 2.0	6.1 ± 1.8	0.004
Spleen	10.8 ± 2.8	9.2 ± 2.0	<0.001	11.1 ± 3.3	9.7 ± 2.4	0.002
Portal vein	10.6 ± 3.5	9.6 ± 2.3	<0.001	10.8 ± 3.3	9.1 ± 2.5	<0.001
Aorta	17.7 ± 5.4	14.3 ± 3.6	<0.001	18.7 ± 5.5	16.0 ± 4.2	<0.001
Kidney	12.0 ± 3.9	10.4 ± 2.5	0.004	12.8 ± 3.7	10.8 ± 2.7	<0.001
CNR						
Liver	2.8 ± 1.3	2.2 ± 1.4	0.012	2.8 ± 1.8	2.4 ± 1.6	0.074
Pancreas	3.7 ± 2.8	2.8 ± 1.6	<0.001	4.3 ± 2.2	3.2 ± 2.0	<0.001
Spleen	6.2 ± 3.2	4.7 ± 2.3	0.002	6.8 ± 3.5	5.7 ± 2.4	0.016
Portal vein	8.6 ± 3.6	6.6 ± 4.2	0.003	10.8 ± 7.7	7.4 ± 2.9	<0.001
Aorta	15.7 ± 6.0	12.0 ± 4.2	<0.001	16.6 ± 6.1	14.1 ± 4.6	0.002
Kidney	9.2 ± 3.8	7.1 ± 2.5	<0.001	10.5 ± 4.1	8.3 ± 3.1	<0.001

SNR, signal-to-noise ratio; CNR, contrast-to-noise ratio.

**Table 6:** Diagnostic performance in all the patients and in the two groups.

		Cyst		Hemangioma		Malignancy	
		Sensitivity (%)	Specificity (%)	Sensitivity (%)	Specificity (%)	Sensitivity (%)	Specificity (%)
Reader 1	Total	91.0	99.3	80.0	99.2	100	99.5
	Group A	87.5	100	90.0	99.0	100	99.5
	Group B	95.2	98.7	73.3	99.5	100	99.5
Reader 2	Total	85.8	99.7	80.0	99.7	94.3	99.7
	Group A	88.9	99.3	100	99.5	100	99.5
	Group B	82.3	100	66.7	100	90.3	100

## Data Availability

The data presented in this study are available on request from the corresponding author. The data are not publicly available due to privacy or ethical concerns.

## References

[1] Isaka Y., Hayashi H., Aonuma K., Horio M., Terada Y., Doi K., Fujigaki Y., Yasuda H., Sato T., Fujikura T. (2020). Guideline on the use of iodinated contrast media in patients with kidney disease 2018. Clin. Exp. Nephrol..

[2] American College of Radiology (2020). ACR Manual on Contrast Media.

[3] European Society of Urogenital Radiology (2018). ESUR Guidelines on Contrast Agents v10.0.

[4] Katzberg R.W., Newhouse J.H. (2010). Intravenous Contrast Medium–induced Nephrotoxicity: Is the Medical Risk Really as Great as We Have Come to Believe?. Radiology.

[5] Katzberg R.W., Lamba R. (2009). Contrast-Induced Nephropathy after Intravenous Administration: Fact or Fiction?. Radiol. Clin. N. Am..

[6] Weisbord S.D., Mor M.K., Resnick A.L., Hartwig K.C., Palevsky P.M., Fine M.J. (2008). Incidence and Outcomes of Contrast-Induced AKI Following Computed Tomography. Clin. J. Am. Soc. Nephrol..

[7] Davenport M.S., Khalatbari S., Dillman J.R., Cohan R.H., Caoili E.M., Ellis J.H. (2013). Contrast Material–induced Nephrotoxicity and Intravenous Low-Osmolality Iodinated Contrast Material. Radiology.

[8] Lencioni R., Fattori R., Morana G., Stacul F., The Italian Observational Study Panel (2010). Contrast-induced nephropathy in patients undergoing computed tomography (CONNECT)—A clinical problem in daily practice? A multicenter observational study. Acta Radiol..

[9] Chua H.-R., Low S., Murali T.M., Wong E.T.-Y., He H.-D., Teo B.-W., Thian Y.-L., Akalya K., Vathsala A. (2021). Cumulative iodinated contrast exposure for computed tomography during acute kidney injury and major adverse kidney events. Eur. Radiol..

[10] From A.M., Bartholmai B., Williams A.W., Cha S.S., McDonald F.S. (2008). Mortality Associated With Nephropathy After Radiographic Contrast Exposure. Mayo Clin. Proc..

[11] Nyman U., Almén T., Aspelin P., Hellström M., Kristiansson M., Sterner G. (2005). Contrast-medium-induced nephropathy correlated to the ratio between dose in gram iodine and estimated gfr in ml/min. Acta Radiol..

[12] Limbruno U., Picchi A., Micheli A., Calabria P., Cortese B., Brizi G., Severi S., De Caterina R. (2014). Refining the assessment of contrast-induced acute kidney injury: The load-to-damage relationship. J. Cardiovasc. Med..

[13] Morcos S. (2009). Contrast-induced nephropathy: Are there differences between low osmolar and iso-osmolar iodinated contrast media?. Clin. Radiol..

[14] Paparo F., Garello I., Bacigalupo L., Marziano A., Pregliasco A.G., Rollandi L., Puppo C., Mattioli F., Puntoni M., Rollandi G.A. (2014). CT of the abdomen: Degree and quality of enhancement obtained with two concentrations of the same iodinated contrast medium with fixed iodine delivery rate and total iodine load. Eur. J. Radiol..

[15] Matoba M., Kitadate M., Kondou T., Yokota H., Tonami H. (2009). Depiction of Hypervascular Hepatocellular Carcinoma With 64-MDCT: Comparison of Moderate- and High-Concentration Contrast Material with and without Saline Flush. Am. J. Roentgenol..

[16] Awai K., Takada K., Onishi H., Hori S. (2002). Aortic and Hepatic Enhancement and Tumor-to-Liver Contrast: Analysis of the Effect of Different Concentrations of Contrast Material at Multi–Detector Row Helical CT. Radiology.

[17] Hänninen E.L., Vogl T.J., Felfe R., Pegios W., Balzer J., Clauss W., Felix R. (2000). Detection of Focal Liver Lesions at Biphasic Spiral CT: Randomized Double-Blind Study of the Effect of Iodine Concentration in Contrast Materials. Radiology.

[18] Furuta A., Ito K., Fujita T., Koike S., Shimizu A., Matsunaga N. (2004). Hepatic Enhancement in Multiphasic Contrast-Enhanced MDCT: Comparison of High- and Low-Iodine-Concentration Contrast Medium in Same Patients with Chronic Liver Disease. Am. J. Roentgenol..

[19] Kim T., Murakami T., Takahashi S., Okada A., Hori M., Narumi Y., Nakamura H. (1999). Pancreatic CT Imaging: Effects of Different Injection Rates and Doses of Contrast Material. Radiology.

[20] Hwang I., Cho J.Y., Kim S.Y., Oh S.-J., Ku J.H., Lee J., Kim S.H. (2015). Low tube voltage computed tomography urography using low-concentration contrast media: Comparison of image quality in conventional computed tomography urography. Eur. J. Radiol..

[21] Kim S.Y., Cho J.Y., Lee J., Hwang S.I., Moon M.H., Lee E.J., Hong S.S., Kim C.K., Kim K.A., Bin Park S. (2018). Low-Tube-Voltage CT Urography Using Low-Concentration-Iodine Contrast Media and Iterative Reconstruction: A Multi-Institutional Randomized Controlled Trial for Comparison with Conventional CT Urography. Korean J. Radiol..

[22] Botsikas D., Barnaure I., Terraz S., Becker C.D., Kalovidouri A., Montet X. (2016). Value of liver computed tomography with iodixanol 270, 80 kVp and iterative reconstruction. World J. Radiol..

[23] Ichikawa S., Motosugi U., Shimizu T., Kromrey M.L., Aikawa Y., Tamada D., Onishi H. (2021). Diagnostic performance and image quality of low-tube voltage and low-contrast medium dose protocol with hybrid iterative reconstruction for hepatic dynamic CT. Br. J. Radiol..

[24] Taguchi N., Oda S., Utsunomiya D., Funama Y., Nakaura T., Imuta M., Yamamura S., Yuki H., Kidoh M., Hirata K. (2017). Using 80 kVp on a 320-row scanner for hepatic multiphasic CT reduces the contrast dose by 50 % in patients at risk for contrast-induced nephropathy. Eur. Radiol..

[25] Zhang H., Ma Y., Lyu J., Yang Y., Yuan W., Song Z. (2017). Low kV and Low Concentration Contrast Agent with Iterative Reconstruction of Computed Tomography (CT) Coronary Angiography: A Preliminary Study. Med Sci. Monit..

[26] Marcus R.P., Koerner E., Aydin R.C., Zinsser D., Finke T., Cyron C.J., Bamberg F., Nikolaou K., Notohamiprodjo M. (2017). The evolution of radiation dose over time: Measurement of a patient cohort undergoing whole-body examinations on three computer tomography generations. Eur. J. Radiol..

[27] Wichmann J.L., Hardie A.D., Schoepf U.J., Felmly L.M., Perry J.D., Varga-Szemes A., Mangold S., Caruso D., Canstein C., Vogl T.J. (2017). Single- and dual-energy CT of the abdomen: Comparison of radiation dose and image quality of 2nd and 3rd generation dual-source CT. Eur. Radiol..

[28] Park C., Gruber-Rouh T., Leithner D., Zierden A., Albrecht M.H., Wichmann J.L., Bodelle B., Elsabaie M., Scholtz J.-E., Kaup M. (2016). Single-source chest-abdomen-pelvis cancer staging on a third generation dual-source CT system: Comparison of automated tube potential selection to second generation dual-source CT. Cancer Imaging.

[29] Scholtz J.E., Wichmann J.L., Hüsers K., Beeres M., Nour-Eldin N.E.A., Frellesen C., Vogl T.J., Lehnert T. (2015). Automated tube voltage adaptation in combination with advanced modeled iterative reconstruction in thoracoabdominal third-generation 192-slice dual-source computed tomography: Effects on image quality and radiation dose. Acad. Radiol..

[30] Lee K.H., Lee J.M., Moon S.K., Baek J.H., Park J.H., Flohr T.G., Kim K.W., Kim S.J., Han J.K., Choi B.I. (2012). Attenuation-based Automatic Tube Voltage Selection and Tube Current Modulation for Dose Reduction at Contrast-enhanced Liver CT. Radiology.

[31] Mayer C., Meyer M., Fink C., Schmidt B., Sedlmair M., Schoenberg S.O., Henzler T. (2014). Potential for Radiation Dose Savings in Abdominal and Chest CT Using Automatic Tube Voltage Selection in Combination With Automatic Tube Current Modulation. Am. J. Roentgenol..

[32] Nakayama Y., Awai K., Funama Y., Hatemura M., Imuta M., Nakaura T., Ryu D., Morishita S., Sultana S., Sato N. (2005). Abdominal CT with Low Tube Voltage: Preliminary Observations about Radiation Dose, Contrast Enhancement, Image Quality, and Noise 1. Radiology.

[33] Araki K., Yoshizako T., Yoshida R., Tada K., Kitagaki H. (2018). Low-voltage (80-kVp) abdominopelvic computed tomography allows 60% contrast dose reduction in patients at risk of contrast-induced nephropathy. Clin. Imaging.

[34] Nakaura T., Nakamura S., Maruyama N., Funama Y., Awai K., Harada K., Uemura S., Yamashita Y. (2012). Low Contrast Agent and Radiation Dose Protocol for Hepatic Dynamic CT of Thin Adults at 256–Detector Row CT: Effect of Low Tube Voltage and Hybrid Iterative Reconstruction Algorithm on Image Quality. Radiology.

